# Gender and Uveitis

**DOI:** 10.1155/2014/818070

**Published:** 2014-07-22

**Authors:** Chi-Chao Chan, Debra A. Goldstein, Janet L. Davis, H. Nida Sen

**Affiliations:** ^1^Laboratory of Immunology, National Eye Institute, Bethesda, MD, USA; ^2^Department of Ophthalmology, Northwestern Feinberg School of Medicine, Chicago, IL, USA; ^3^Bascom Palmer Eye Institute, University of Miami, Miami, FL, USA

Sex differences in medicine include sex-specific diseases occurring only in one sex and sex-related diseases that are more common to one sex. Indeed differences in incidence, presentation, and course of disease between males and females are common. Eye disease is no exception. According to the WHO website, “In every region of the world and at all ages, females have a significantly higher risk of being visually impaired.” WHO estimates that globally there are approximately 314 million people with visual impairment and that women account for more than 64.5%. Even adjusted for age, the overall odds ratio of blind women to men is 1.43, representing a range from 1.39 in Africa to 1.63 in industrialized countries [1]. Gender-based differences refer to hormonal changes in menstrual cycles, pregnancy, menopause, disease susceptibility, and other anatomic or physiologic differences between women and men. Although the exact mechanisms are not fully understood, recent research demonstrates a link between sex hormones and microbial exposure in which microbiome can trigger testosterone-dependent protection from autoimmunity in the nonobese diabetic (NOD) mouse model [2, 3]. Sex hormones, X-chromosome-related effects, and epigenetic and environmental factors also affect activation and differentiation of different immune cells that play important roles in infectious and autoimmune diseases [4].

The differences of ocular diseases between the sexes are nowhere more apparent than in the field of ocular inflammation. Females as a gender group have heightened immune responses not only to foreign antigens but also to self-antigens. Thus there is a greater preponderance of autoimmune disorders including noninfectious uveitis in women than in men. Recently, the Pacific Ocular Inflammation Study reported that, of 217 061 eligible patients, 872 were identified using International Classification of Diseases, Ninth Revision codes, and 224 cases of uveitis were confirmed. The overall uveitis incidence rate was 24.9 cases per 100000 person-years. The annual prevalence rates for 2006 and 2007 were 57.5 and 58.0 per 100000 persons, respectively. No difference in incidence rate was found by sex (*P* = 0.63), but female patients had a higher prevalence (*P* = 0.008) [5]. This special issue attempts to identify gender- and sex-based differences in various uveitides: infectious and noninfectious autoimmune uveitis, for example, multiple sclerosis in young and middle-aged women, juvenile idiopathic arthritis in girls, and syphilitic uveitis in HIV infected patients in males. Clinical manifestations and courses may appear differently between female and male patients in certain uveitides. Gender-based differences in uveitis should be also considered in care and treatment of the diseases, as well as the underlying genetic background and physical and social environment.

*Chi-Chao Chan* *Chi-Chao Chan* *Debra A. Goldstein* *Debra A. Goldstein* *Janet L. Davis* *Janet L. Davis* *H. Nida Sen* *H. Nida Sen*
